# Association between dietary glycemic index and non-alcoholic fatty liver disease in patients with type 2 diabetes mellitus

**DOI:** 10.3389/fendo.2023.1228072

**Published:** 2023-08-22

**Authors:** Marieh Salavatizadeh, Samira Soltanieh, Amirhossein Ataei Kachouei, Zahra Abdollahi Fallahi, Hamed Kord-Varkaneh, Hossein Poustchi, Asieh Mansour, Mohammad E. Khamseh, Fariba Alaei-Shahmiri, Heitor O. Santos, Azita Hekmatdoost

**Affiliations:** ^1^ Department of Clinical Nutrition and Dietetics, Faculty of Nutrition and Food Technology, Shahid Beheshti University of Medical Sciences, Tehran, Iran; ^2^ Endocrine Research Center, Institute of Endocrinology and Metabolism, Iran University of Medical Sciences, Tehran, Iran; ^3^ Department of Clinical Nutrition, School of Nutrition & Food Science, Isfahan University of Medical Sciences, Isfahan, Iran; ^4^ Science and Research Branch, Islamic Azad University, Tehran, Iran; ^5^ Department of Nutrition and Food Hygiene, School of Medicine, Nutrition Health Research Center, Hamadan University of Medical Sciences, Hamadan, Iran; ^6^ Liver and Pancreatobiliary Diseases Research Center, Digestive Diseases Research Institute, Shariati Hospital, Tehran University of Medical Sciences, Tehran, Iran; ^7^ Endocrinology and Metabolism Research Center, Endocrinology and Metabolism Clinical Sciences Institute, Tehran University of Medical Sciences, Tehran, Iran; ^8^ School of Medicine, Federal University of Uberlandia (UFU), Uberlandia, Minas Gerais, Brazil

**Keywords:** carbohydrate, diet, NAFLD, diabetes, insulin resistance

## Abstract

**Objective:**

Managing dietary glycemic index (GI) deserves further attention in the interplay between non-alcoholic fatty liver disease (NAFLD) and type 2 diabetes mellitus (T2DM). This study aimed to evaluate the relationship between dietary GI and the odds of NAFLD in patients with T2DM.

**Methods:**

A cross-sectional study was carried out between April 2021 and February 2022, including 200 participants with T2DM aged 18-70 years, of which 133 had NAFLD and 67 were in the non-NAFLD group. Cardiometabolic parameters were analyzed using standard biochemical kits and dietary intake was assessed using a validated food frequency questionnaire. Binary logistic regression was applied to explore odds ratios (ORs) and 95% confidence intervals (CIs) for NAFLD according to tertiles of dietary GI.

**Results:**

Highest vs. lowest tertile (< 57 vs. > 60.89) of energy-adjusted GI was not associated with the odds of having NAFLD (OR 1.25, 95% CI = 0.6-2.57; P-trend = 0.54) in the crude model. However, there was an OR of 3.24 (95% CI = 1.03-10.15) accompanied by a significant trend (P-trend = 0.04) after full control for potential confounders (age, gender, smoking status, duration of diabetes, physical activity, waist circumference, HbA1c, triglycerides, total cholesterol, dietary intake of total carbohydrates, simple carbohydrates, fat, and protein).

**Conclusion:**

High dietary GI is associated with increased odds of NAFLD in subjects with T2DM. However, interventional and longitudinal cohort studies are required to confirm these findings.

## 1 Introduction

Diabetes is one of the main health issues worldwide with an estimated prevalence of 9.3% (463 million), reaching 10.2% (578 million) by 2030. Within this population, almost 90% have type 2 diabetes mellitus (T2DM) (1). It is a metabolic disorder characterized by hyperglycemia resulting from either insufficient insulin secretion or ineffective insulin action (2). Deficit in insulin secretion and high insulin resistance increase the lipase enzyme activity gradually leading to impairment of free fatty acid (FFA) metabolism and exceeding the amount of FFA beyond the liver’s ability to oxidize (3, 4). Over-accumulation of these FFAs in the form of hepatic triglycerides (TG) results in a phenomenon known as nonalcoholic fatty liver disease (NAFLD) (4).

NAFLD occurs in 75% of T2DM patients (5). In contrast, T2DM is present in nearly a quarter of patients with NAFLD, and approximately half of the non-alcoholic steatohepatitis patients (5). This coexistence occurs due to shared pathogenic abnormalities caused by excess adipose tissue and insulin resistance (6). Furthermore, the concomitant presence of T2DM and NAFLD has been proposed to be associated with higher overall mortality and mortality related to liver and cardiovascular diseases (3, 7). Therefore, it seems crucial to detect the contributing factors of this co-occurrence.

Nutrition is known as a major modifiable environmental factor in the development and management of NAFLD (8), whose disease when left untreated increases the risk of hepatic and extra-hepatic cancers (e.g., lung, breast, gynecologic, and urinary system cancers) (9–11). The glycemic index (GI) has been studied extensively as a contributing factor for T2DM (12), as well as for alarming diseases such as cancer (13). GI is defined as the ratio between the area under the glucose response curve after consumption of 50 g carbohydrates from a test food and the area under the curve after consumption of 50 g reference food (either white bread or glucose) (14, 15). Food sources of carbohydrates that are digested, absorbed, and metabolized quickly are referred to as high GI foods (16). On the other hand, food sources of carbohydrates with slow digestion, absorption, and metabolism are considered low GI foods (16).

Several studies suggest that a low GI diet may reduce insulin resistance (17–19). In a meta-analysis of cohort studies, diets higher in GI significantly increased the risk of T2DM in healthy individuals regardless of dietary fiber (12). In addition, a meta-analysis of randomized clinical trials (RCTs) suggested anti-inflammatory properties of diets with low overall dietary GI (20). Interestingly, a low GI Mediterranean diet decreased NAFLD scores in an RCT (21). Conversely, diets with high total dietary GI could increase indicators of systemic inflammation considered to be key factors of the NAFLD risk in individuals with T2DM (22, 23).

Therefore, individuals with T2DM need to specifically be considered in the evaluation of potential dietary prevention strategies for NAFLD. Correspondingly, to the best of our knowledge, the association between dietary GI and the development of NAFLD in individuals with T2DM has not yet been examined. Thus, the present study was conducted to assess possible associations between dietary GI and odds of NAFLD in individuals with T2DM.

## 2 Materials and methods

### 2.1 Study participants

This cross-sectional study was carried out between April 2021 and February 2022 on patients with T2DM aged between18-70 years from the diabetes clinic affiliated with the Institute of Diabetes and Metabolism, Iran University of Medical Sciences, Tehran, Iran.

**Figure 1:**
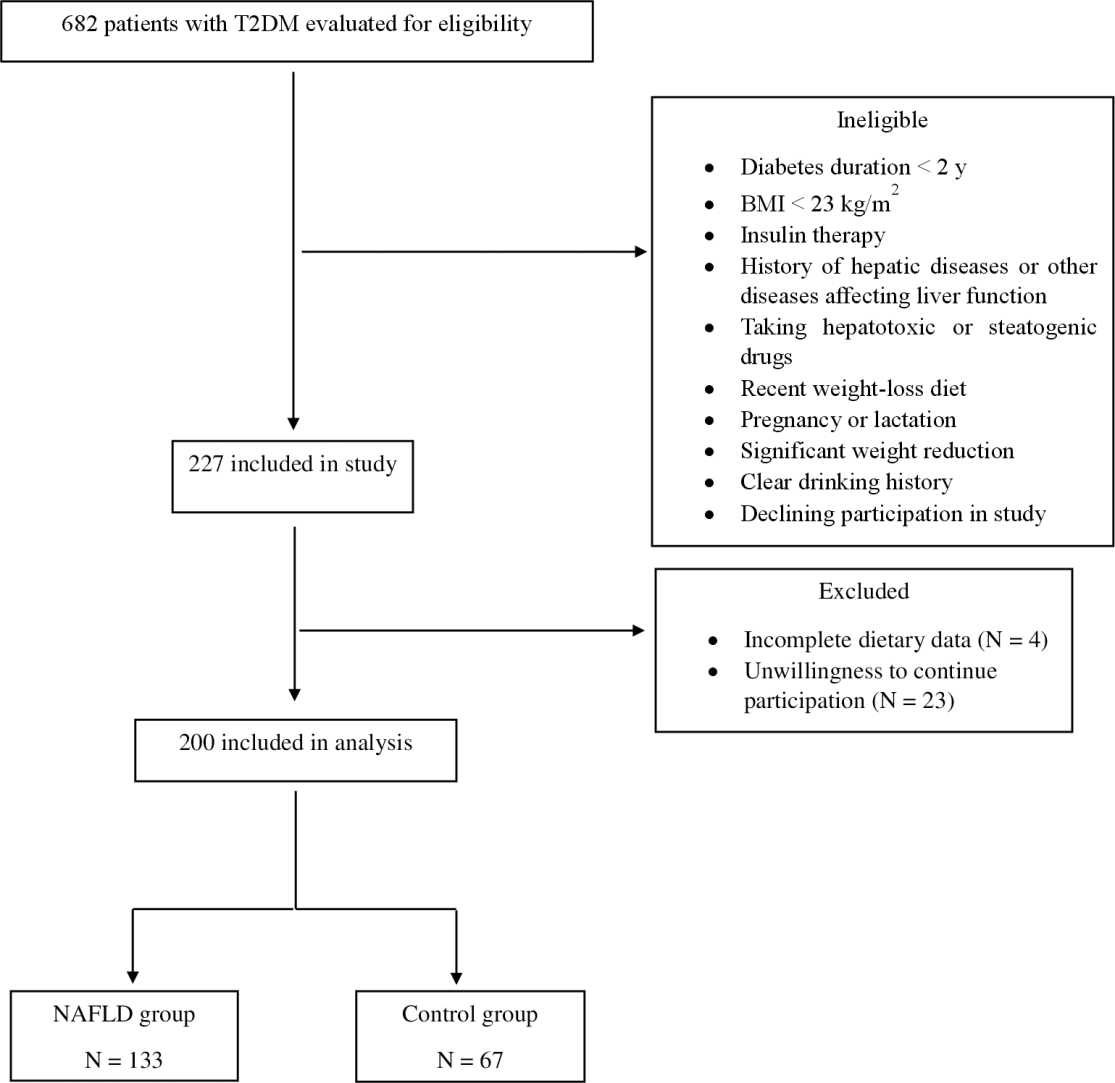
Flow chart of participation.

Patients with a history of any type of pathologically confirmed cancer, chemotherapy or radiotherapy (due to cancer), drug use, chronic inflammatory disease, heart failure, myocardial infarction, and kidney disease were not included in the study. Moreover, participants were excluded upon recently weight-loss diet, taking weight-loss medications, pregnancy, lactation, more than 10% weight reduction during the last 6 months, history of acute and chronic liver diseases (hepatitis, autoimmune disease, biliary disease, hereditary disorders of the liver including Wilson’s disease) and hemochromatosis, and using toxins or drugs affecting the liver such as NSAIDs, anti-inflammatory drugs, etc. Participants with a clear drinking history (≥21 units/week in men and ≥14 units/week in women) were also excluded from the study. Patients who were on insulin therapy were not included. Therefore, participants took only oral hypoglycemic agents for diabetes control. Body mass index (BMI) ≥ 23 kg/m^2^ was an inclusion criterion for all subjects. The participant selection flowchart is indicated in **Figure 1**. In order to detect and quantify liver steatosis, we used the controlled attenuation parameter (CAP) determined by transient elastography (TE) using the FibroScan®, equipped with M and XL probes. In the present study, the cut-off value for diagnosing hepatic steatosis was the CAP value > 270 dB/m (24). Data on demographic characteristics were collected by means of a standard questionnaire by trained interviewers.

The study protocol was approved by the Ethics Committee of the Shahid Beheshti University of Medical Sciences (NO: IR.SBMU.NNFTRI.REC.1399.061). Eligible volunteers were selected by the use of the consecutive-sampling method and provided informed written consent, prior to study commencement.

### 2.2 Sample size calculation

The sample size was based on a previous study (25) with SGOT levels of 14 ± 7 and 11 ± 3 IU/L for patients suffering from T2DM with and without NAFLD, respectively. At 95% CI and 80% power of the study, a sample of at least 56 subjects in each group was estimated using the following formula:


(1) $$\frac{{\left({\text{Z}}_{\alpha }+{\text{Z}}_{\text{B}}\right)}^{2}\left({\text{S}}_{1}^{2}+{\text{S}}_{2}^{2}\right)}{{\left({\bar{\text{x}}}_{1}-{\bar{\text{x}}}_{2}\right)}^{2}}$$

### 2.3 Anthropometric and physical activity assessments

Subjects’ body mass (kg) was evaluated unshod and in light clothing using a digital scale (Seca, Germany) to the nearest 100 g. Height was measured without shoes in a standing position using a fixed tape measure to the nearest 0.5 cm. Finally, BMI was calculated by dividing weight (kg) by the square of height (meters).

The International Physical Activity Questionnaire (IPAQ) short form was applied to assess subjects’ physical activity during the last 7 days and was expressed as the metabolic equivalent task (MET)-min/week (26). The validity and reliability of this questionnaire have previously been evaluated in Iranian adult women. Blood pressure was measured for all participants using an automatic sphygmomanometer (OMRON, Germany) on the left arm in a sitting position after a rest of at least 10 minutes. By selecting an appropriate cuff size and preventing patients from speaking during measurements, errors were avoided.

### 2.4 Laboratory measurements

Venous blood samples were collected after 10-12 hours of overnight fasting. The enzymatic colorimetric method was applied to determine fasting blood sugar (FBS) levels. Enzymatic assays were performed to measure the serum levels of TG, total cholesterol (TC), and high-density lipoprotein (HDL) by the use of standard biochemical kits (Pars Azmun Co., Iran) with between- and within-run coefficient of variations<6.2%. Low-density lipoprotein (LDL) was calculated through the use of the modified version of the Friedewald equation (27). Roche Diagnostics kits (Roche Cobas 6000 analyzer) were used to measure serum insulin levels by means of the ECLIA method. HOMA-IR (Homeostatic Model Assessment for Insulin Resistance) was calculated by the following equation: [fasting insulin (μU/mL)×fasting glucose (mmol/L)]/22.5  (28). QUICKI (Quantitative Insulin Sensitivity Check Index) was computed as 1/[log(fasting insulin in μU/mL)+ log (fasting glucose in mg/dL )] (29). TyG (Triglyceride and glucose) index was determined as Ln [TG (mg/dL) ×FGS (mg/dL)/2]  (30).

### 2.5 Dietary intake assessment

Dietary intakes of participants over the past year were examined using a validated 147-item semi-quantitative food frequency questionnaire (FFQ) (31). An expert registered dietitian was totally unaware of the participants’ condition (in terms of having NAFLD) and applied the FFQ via face-to-face interviews. Each participant reported the average intake of different food items during the preceding year on a daily, weekly, or monthly basis which was converted to grams per day using household measures (32). Subsequently, daily nutrients and energy intakes were determined using Nutritionist 4 software (First Databank Inc., Hearst Corp., San Bruno, CA, USA) modified for Iranian foods.

### 2.6 Calculation of the dietary glycemic index

The GI of every consumed food item was calculated by using the following formula: (GI × available carbohydrate per one gram of food × gram per day of food)/total available carbohydrate, where total available carbohydrates were determined as daily consumed carbohydrates minus total fiber consumed daily (33). The daily GI values of each carbohydrate-containing food and beverage were determined based on the International Glycemic Index Table 2008 (34). The Iranian GI table was used for calculating the GI for some Iranian food which did not found in the international table (35). Moreover, for food items whose GI was not available on these tables, physically and chemically similar foods were used. Glucose was applied as a reference food to determine the GI variable in this study. For composite mixed meals, the GI values were estimated based on the GIs of individual food components (35). The overall dietary GI was calculated by summing the GIs for all foods consumed in the diet. Dietary glycemic index was adjusted for total energy intake by the residual method (36).

### 2.7 Statistical analysis

The results are presented as mean ± standard deviation (SD) for continuous data and percent for qualitative data. The normality of the data distribution was checked using the Kolmogorov–Smirnov test and the histogram chart. The independent Student’s t-test and Mann–Whitney test were used to compare general characteristics with normal and abnormal distributions between the study groups, respectively. The X^2^ test was applied for qualitative variables.

Participants were categorized into tertiles of dietary GI. Comparisons for general characteristics, biochemical parameters, and dietary intakes across tertiles of dietary GI were performed by applying the Kruskal-Wallis test and analysis of covariance (ANOVA) for continuous variables and the X^2^ test for categorical variables. Binary logistic regression in different models was used to explore the association between dietary GI and NAFLD in patients with T2DM. In all analyses, the first tertile of dietary GI was regarded as the reference category. A broad range of confounders was controlled to examine whether the association was independent of them. We used a stepwise (forward) selection procedure for modeling, and variables entered as confounders to the logistic models based on the following confounding criteria: 1) differed between the NAFLD and non-NAFLD groups, 2) associated with the exposure of interest (dietary GI) and 3) not an intermediate the pathway. Moreover, all covariates were assessed for multicollinearity. The exponential of betas was interpreted as Odds Ratios. The tertiles of the dietary GI were included as an ordinal variable in the model to examine the overall trend of ORs. All statistical analyses were performed using SPSS (SPSS Inc., version 25). P-values less than 0.05 were considered significant.

## 3 Results

In this study, 200 subjects were enrolled and divided into two groups: NAFLD and non-NAFLD groups, on the basis of CAP score. Those with a CAP score > 270 dB/m were regarded as the NAFLD group whereas participants in the non-NAFLD group had a CAP score ≤ 270 dB/m. Comparisons for characteristics between NAFLD and non-NAFLD groups are presented in **Table 1**. Participants had a mean age of 52.21 ± 9.27 years. Women accounted for 58.64% of the individuals with NAFLD, while 44.77% of the non-NAFLD group were women. Compared to the non-NAFLD group, patients with NAFLD significantly had higher values of BMI (*p*<0.001), WC (*p*<0.001), TC (*p*=0.002), TG (*p*=0.005), LDL (*p*=0.005), SGPT (*p*=0.02), SGOT (*p*=0.04), HbA1c (*p*=0.01), and TyG index (*p*=0.02), as well as HOMA-IR (P<0.001) while QUICKI was significantly lower in NAFLD group (P<0.001). However, there was no statistically significant difference considering smoking status, duration of diabetes, blood pressure, physical activity, FBS, HDL, and dietary GI.

**Table 1:** General characteristics of enrolled subjects^‡^.

Variable	All participants	NAFLD group (n = 133)	non-NAFLD group (n = 67)	P-value^†^
Age (year)	52.21 ± 9.27	52.19 ± 9.06	52.24 ± 9.75	0.84
Sex (Female, %)	54	58.64	44.77	0.07
Smoking (%)	17.5	18.04	16.41	0.84
Duration of diabetes (year)	8.89 ± 5.82	8 ± 5.26	10 ± 6.77	0.27
Body mass (kg)	78.49 ± 14.4	81.4 ± 15.08	72.7 ± 10.91	<0.001
BMI (kg/m^2^)	28.76 ± 4.27	30.07 ± 4.06	26.17 ± 3.42	<0.001
WC (cm)	**Men:** 101.86 ± 10.89 **Women:** 99.35 ± 10.63	105.84 ± 11.35 101.95 ± 10.3	95.94 ± 6.81 92.6 ± 8.36	<0.001 <0.001
SBP (mmHg)	123.67 ± 15.04	123 ± 14.55	125 ± 16.03	0.85
DBP (mmHg)	77.03 ± 10.02	78 ± 10.42	75 ± 9.02	0.11
Physical activity (MET-min/week)	879.55 ± 1488.17	950.83 ± 1757.85	738.06 ± 683.27	0.37
FBS (mg/dL)	149.95 ± 59.61	150.53 ± 59.22	148.79 ± 60.81	0.36
TC (mg/dL)	146.6 ± 47.95	153.68 ± 51.75	132.52 ± 35.69	0.002
TG (mg/dL)	167.16 ± 160.17	179.98 ± 173.01	141.69 ± 128.45	0.005
HDL (mg/dL)	**Men:** 45.66 ± 12.11 **Women:** 52.58 ± 12.61	46.02 ± 12.75 51.87 ± 11.85	45.16 ± 11.32 54.43 ± 14.45	0.84 0.56
LDL (mg/dL)	73.74 ± 26.89	77.54 ± 27.77	66.37 ± 23.6	0.005
SGPT (IU/L)	18.86 ± 9.76	20.05 ± 10.62	16.49 ± 7.28	0.02
SGOT (IU/L)	20.52 ± 8.94	21.38 ± 9.05	18.82 ± 8.53	0.04
HbA1c (%)	7.72 ± 1.79	7.92 ± 1.85	7.33 ± 1.64	0.01
Insulin (μU/mL)	7.75 ± 5.86	8.77 ± 6.56	5.73 ± 3.36	<0.001
HOMA-IR	2.78 ± 2.42	3.23 ± 2.79	1.89 ± 0.94	<0.001
QUICKI	0.34 ± 0.03	0.33 ± 0.03	0.35 ± 0.02	<0.001
TyG index	3.98 ± 0.31	4.02 ± 0.31	3.91 ± 0.3	0.02
Energy-adjusted GI	58.59 ± 5.21	58.51 ± 5.43	58.73 ± 4.77	0.78

^‡^Variables are mean ± SD, unless indicated.

^†^Independent Student’s t-test and Mann–Whitney test were used to compare continuous variables with normal and abnormal distributions, respectively. Chi-square test was used to compare qualitative variables.

BMI, Body mass index; WC, waist circumference; SBP, systolic blood pressure; DBP, diastolic blood pressure; MET, metabolic equivalents; FBS, fasting blood glucose; TC, total cholesterol; TG, triglyceride; HDL, high-density lipoprotein-cholesterol; LDL, low-density lipoprotein-cholesterol; SGPT, serum glutamate pyruvate transaminase; SGOT, serum glutamic-oxaloacetic transaminase; HbA1c, hemoglobin A1c; HOMA - IR, Homeostatic Model Assessment of Insulin Resistance; QUICKI, quantitative insulin-sensitivity check index; TyG index, Triglyceride-glucose index; GI, glycemic index.

General characteristics of the participants are represented in **Table 2** based on dietary GI tertiles. Age (*p*=0.002), duration of diabetes (*p*=0.005), body mass (*p*=0.03), HDL among men (*p*=0.001), SGOT (*p*=0.03), insulin (*p*<0.001), HOMA-IR (*p*<0.001), and QUICKI (*p*<0.001) were significantly different between dietary GI tertiles. However, there was no significant difference in the distribution of sex, smoking status, BMI, blood pressure, physical activity, TyG index, FBS, and HbA1c.

**Table 2:** General characteristics of subjects across tertiles of energy-adjusted dietary glycemic index^‡^.

Variable	Tertile 1 (< 57)	Tertile 2 (57 to 60.89)	Tertile 3 (> 60.89)	P-value^†^
Number of participants	66	67	67	
Age (year)	55.45 ± 8.49	50.36 ± 8.29	50.85 ± 10.19	0.002
Sex (Female, %)	41	36	31	0.18
Smoking (%)	10	13	12	0.8
Duration of diabetes (year)	10.75 ± 7.14	7.63 ± 3.98	8.33 ± 5.54	0.005
Body mass (kg)	75.73 ± 13.16	77.72 ± 13.75	81.99 ± 15.64	0.03
BMI (kg/m^2^)	28.21 ± 3.6	28.66 ± 4.69	29.41 ± 4.41	0.35
WC (cm)	**Men:** 100.12 ± 7.65 **Women:** 101.12 ± 12.18	101.21 ± 12.05 97.25 ± 9.39	103.63 ± 11.76 99.46 ± 9.65	0.43 0.28
SBP (mmHg)	123.68 ± 17.25	122.01 ± 14.9	125.3 ± 12.71	0.45
DBP (mmHg)	76.45 ± 10.55	77.72 ± 9.54	76.9 ± 10.07	0.53
Physical activity (MET-min/week)	523.07 ± 189.01	775.57 ± 865.15	1334.7 ± 2354.89	0.12
FBS (mg/dL)	155.77 ± 61.31	138.51 ± 43.46	155.66 ± 70.17	0.47
TC (mg/dL)	147.62 ± 41.40	148.28 ± 39.90	143.90 ± 60.35	0.33
TG (mg/dL)	161.52 ± 129.83	152.3 ± 103.96	187.57 ± 221.96	0.42
HDL (mg/dL)	**Men:** 47.16 ± 13.84 **Women:** 54.63 ± 12.4	50.63 ± 11.53 51.36 ± 15.07	40.18 ± 8.95 51.29 ± 9.39	0.001 0.42
LDL (mg/dL)	77.78 ± 28.56	74.11 ± 26.42	69.27 ± 25.3	0.19
SGPT (IU/L)	18.52 ± 10.7	18.57 ± 9.5	18.57 ± 9.15	0.58
SGOT (IU/L)	18.97 ± 9.31	19.75 ± 6.51	22.82 ± 10.24	0.03
HbA1c (%)	7.77 ± 1.64	7.47 ± 1.54	7.93 ± 2.14	0.32
Insulin (μU/mL)	5.73 ± 3.39	7.51 ± 4.39	9.98 ± 7.99	<0.001
HOMA-IR	2.08 ± 1.33	2.49 ± 1.67	3.77 ± 3.39	<0.001
QUICKI	0.35 ± 0.03	0.34 ± 0.02	0.33 ± 0.03	<0.001
TyG index	4 ± 0.3	3.93 ± 0.29	4.02 ± 0.34	0.32

^‡^Variables are mean ± SD, unless indicated. Energy-adjusted dietary glycemic index was calculated using the residual method.

^†^One-way analysis of variance and Kruskal-Wallis tests were used to compare continuous variables with normal and abnormal distributions, respectively. Chi-square test was used to compare qualitative variables.

BMI, Body mass index; WC, waist circumference; SBP, systolic blood pressure; DBP, diastolic blood pressure; MET, metabolic equivalents; FBS, fasting blood sugar; TC, total cholesterol; TG, triglyceride; HDL, high-density lipoprotein-cholesterol; LDL, low-density lipoprotein-cholesterol; SGPT, serum glutamate pyruvate transaminase; SGOT, serum glutamic-oxaloacetic transaminase; HbA1c, hemoglobin A1c; HOMA - IR, Homeostatic Model Assessment of Insulin Resistance; QUICKI, quantitative insulin-sensitivity check index; TyG index, Triglyceride-glucose index.

**Table 3** depicts the food intake distribution stratified by dietary GI tertiles. The energy intake was not significantly different among tertiles. Nevertheless, significant differences in the intake of carbohydrates (*p*=0.03), fruits (*p*=0.001), sweetened beverages (*p*=0.03), dairy products (*p*=0.002), vegetables (*p*<0.001), bread, and legumes (*p*<0.001) were observed in the dietary GI tertiles. A statistically significant difference was seen for fat (*p*=0.005), MUFA (*p*<0.001), and potassium (*p*<0.001) intake between tertiles.

**Table 3:** Dietary intakes of subjects across tertiles of energy-adjusted dietary glycemic index^‡^.

Variable	Tertile 1 (< 57)	Tertile 2 (57 to 60.89)	Tertile 3 (> 60.89)	P-value^†^
Number of participants	66	67	67	
Energy (kcal/day)	2462.96 ± 1058.18	2547.46 ± 1012	2487.26 ± 679.53	0.87
Carbohydrates (% energy intake)	55.15 ± 11.28	58.59 ± 8.05	59.11 ± 8.19	0.03
Protein (% energy intake)	18.74 ± 30.35	14.47 ± 2.54	18.31 ± 32.58	0.62
Fat (% energy intake)	35.16 ± 12.3	30.48 ± 7.29	29.88 ± 10.31	0.005
PUFA (% energy intake)	7.26 ± 3.21	6.64 ± 2.31	6.34 ± 2.09	0.11
MUFA (% energy intake)	11.71 ± 3.34	10.35 ± 2.88	9.7 ± 2.42	<0.001
Fiber (g/1000 kcal)	15.72 ± 4.97	17.18 ± 4.52	17.39 ± 7.02	0.17
Potassium (mg/1000 kcal)	2028.03 ± 498/31	1827.68 ± 303.19	1619.42 ± 259.5	<0.001
Sodium (mg/1000 kcal)	2461.67 ± 1847/39	2187.61 ± 1142.53	6156.89 ± 34119.31	0.32
Fruits (g/1000 kcal)	295.31 ± 149.09	209.37 ± 107.02	145.01 ± 66.36	<0.001
Vegetables (g/1000 kcal)	218.77 ± 122.19	163.78 ± 70.02	136.89 ± 67.38	<0.001
Bread and legumes (g/1000 kcal)	46.3 ± 37.36	81.13 ± 52.84	82.16 ± 58.46	<0.001
Sweetened beverages (% energy intake)	2.15 ± 5.11	2.25 ± 3.7	4.13 ± 10.84	0.03
Dairy products (g/1000 kcal)	175.14 ± 115.29	147.4 ± 87.01	110.43 ± 58.74	0.002

^‡^Variables are mean ± SD. Energy-adjusted dietary glycemic index was calculated using the residual method.

^†^One-way analysis of variance and Kruskal-Wallis tests were used to compare continuous variables with normal and abnormal distributions, respectively.

MUFA, monounsaturated fatty acid; PUFA, polyunsaturated fatty acid.

**Table 4** represents the association between GI and the odds of having NAFLD along with T2DM. According to the crude model, GI tertiles and NAFLD did not correlate significantly. However, after adjustment for potential confounders including age, gender, smoking status, duration of diabetes, physical activity, WC, Hb1Ac, TG, TC, dietary intake of carbohydrates, fat, protein, and simple carbohydrates, there was a significant positive association between GI and the odds of NAFLD (P for trend = 0.04).

**Table 4:** Crude and multivariable-adjusted odds ratios (ORs) and 95% confidence intervals (95% CIs) for NAFLD in patients with T2DM across tertiles of energy-adjusted dietary glycemic index.

		Energy-adjusted dietary glycemic index^‡^			P-trend^†^
		T1 (n = 66) < 57	T2 (n = 67) 57–60.89	T3 (n = 67) > 60.89	
		OR	OR(95 % CI)	OR (95 % CI)	** **
Crude	Overall	1(Ref)1.16	(0.57-2.39)	1.25 (0.6-2.57)	0.54
	**Men**	1(Ref)	(0.44-3.69)	1.86 (0.64-5.25)	0.24
	**Women**	1(Ref)	1.24 (0.45-3.41)	1.01 (0.36-2.82)	0.95
Model 1	**Overall**	1(Ref)	1.61 (0.69 - 3.78)	1.48 (0.62-3.52)	0.37
	**Men**	1(Ref)	1.11 (0.32-3.83)	1.47 (0.43-4.97)	0.53
	**Women**	1(Ref)	2.82 (0.75-10.51)	1.41 (0.38-5.24)	0.59
Model 1	**Overall**	1(Ref)	1.83 (0.75 - 4.47)	1.81 (0.72-4.53)	0.2
	**Men**	1(Ref)	1.02 (0.26-3.92)	1.7 (0.46-6.29)	0.41
	**Women**	1(Ref)	2.7 (0.6-12.07)	1.41 (0.31-6.47)	0.62
Model 3	**Overall**	1(Ref)	2.36 (0.9 - 6.19	3.24 (1.03-10.15)	0.04
	**Men**	1(Ref)	2.17 (0.44-10.65)	4.68 (0.76-28.58)	0.09
	**Women**	1(Ref)	3.32 (0.58-19.07)	2.34 (0.26-20.47)	0.41

Model 1: Adjusted for age, gender, smoking status, duration of diabetes, physical activity, and WC.

Model 2: Adjusted for HbA1c, TG, and TC in addition to the confounders of the first model.

Model 3: Adjusted for dietary intake of carbohydrates (% energy intake), fat (% energy intake), protein (% energy intake), and simple carbohydrates (% energy intake) in addition to the confounders of the second model.

^‡^Energy-adjusted dietary glycemic index was calculated using the residual method.

^†^Binary logistic regression models were employed to obtain odds ratios (ORs) and 95% CIs.

## 4 Discussion

Taken together, this study shows that a higher GI can be associated with NAFLD in patients with T2DM. More specifically, patients in the highest tertile of the dietary GI had 3.24 times increased likelihood of having NAFLD (95% CI: 10.15 - 1.03) compared to those in the first tertile, whose result was observed over full adjustment. On the other hand, we did not observe associations between GI and NAFLD in the other adjusted models, as well as in the crude model. Given that the adjusted model 1 focused on demographic variables (age, sex, smoking status, duration of diabetes, physical activity, in addition to WC as an indicator of obesity) and the adjusted model 2 focused on the addition of traditional metabolic biomarkers (HbA1c, TG, and TC) to the model 1, the inclusion of dietary data from adjustment model 3 (% energy intake of dietary intake of total carbohydrates, simple carbohydrates, fat, and protein, alongside confounders from model 2) seemingly reached statistical significance because it provided a more reliable result after controlling for crucial confounders in nutrition.

Regarding the mechanisms between T2DM and NAFLD, there is a close pathophysiological link between these ailments for which hepatic insulin resistance is likely the central tenet by raising hepatic TG synthesis triggered by fatty acids released from insulin-resistant adipocytes and *De Novo* Lipogenesis, whose latter process consists of elevated glycerol esterification of glycerol upon increased gluconeogenesis and decreased glycogenesis (37, 38). Acutely, in postprandial hyperglycemia, there is an overload of acetyl-CoA release by excess intake of carbohydrates and lipids; acetyl-CoA then enters the citric acid cycle, where the acetyl group is oxidized to carbon dioxide and water, and the released energy produces ATP and free radicals at the same time, which chronic overload may affect the integrity of hepatocytes due to the sharp damage induced by oxidative stress (16, 39).

Our results are of clinical relevance given the relationship between GI and postprandial glycemia, in which higher postprandial glycemia is associated with histological severity in patients with NAFLD (40). Additionally, higher postprandial glycemia is a harbinger of cardiometabolic diseases (e.g., diabetes and heart disease), while low-GI diets portray protection comparable to that seen for whole grain and high fiber intake, as shown by a systematic review of 37 prospective cohort studies (41). Although our study does not infer causation, a meta-analysis of RCTs with interventions >4 weeks detected a decrease of 0.4 HbA1c with low-GI diets compared with higher-GI diets in patients with T1DM or T2DM (n = 457 from 7 studies) (42). Moreover, the review by Parker et al. and Kim supports that low GI can reduce hepatic fat mass and SGOT levels in patients with NAFLD (43).

Apart from the overall results of meta-analyses, well-controlled RCTs must be examined alone to provide expanded specificity. That said, in the GLYNDIET study, a 6-month RCT consisting of 122 patients with overweight and obesity, those who followed a low-GI diet containing moderate amounts of carbohydrates had more efficacy at reducing body weight and controlling glucose and insulin metabolism compared to a high-GI diet containing moderate amounts of carbohydrates and to a low-fat and high-GI diet, in which the 3 diets were isocaloric with energy restriction (44). In contrast, in a 3-moth RCT with a crossover fashion encompassing 19 women with overweight or obesity accompanied by moderate hyperinsulinemia, reducing GI by managing versions of common carbohydrate-rich foods (e.g., breakfast cereals, breads, pasta, and potatoes, and rice) did not enhance body weight, energy intake, and satiety when compared to the condition of high GI (45).

Although low-, moderate-, and high-GI foods have a GI of ≤55 or less, between 56 and 69, and ≥70, respectively (46), in our study, the GI in tertiles 1, 2, and 3 ranged<57, 57 to 60.89, and >60.89, respectively, and thus have some differences compared to the traditional GI classification. At best, this was a reasonable way to categorize the population according to GI, whose tertiles are expected to be closer to the traditional GI classification, but not necessarily the same. The GI theory is convenient for educational purposes, but it should be deciphered and used widely due to its limitations. Correspondingly, the GI has a questionable practical application in the field of clinical nutrition, as carbohydrate sources are commonly combined with different foods, in which little amounts of fat, protein, and fiber can lower the GI of the food markedly. Moreover, the GI concept is paradoxical, while foods with high amounts of monosaccharides have a high GI, and high fiber foods and polysaccharides have a lower GI, fructose is a low GI monosaccharide and some starch sources can have a high GI.

Potato consumption can be a universal example that a high GI food is not necessarily unhealthy and leads to fat gain; conversely, it can be an ally. Although potatoes are classified as a high-GI food, they are sources of potassium and have fewer calories—due to the lower carbohydrate content—compared to traditional food substitutes such as rice, bread, and pasta. Interestingly, while the GI of white potatoes alone is 69-98 (47) and the GI of pasta alone is 43-61 (47, 48), white boiled potatoes have 125 kcal and 20.4 carbohydrates per 100 g [FDC ID: 1102882 (49)], while cooked pasta has 30.7 g of carbohydrates and 157 kcal per 100 g [FDC ID: 1101529 (50)].

In an RCT consisting of healthy participants, daily intake of non-fried potatoes did not affect glycemic markers and was associated with better diet quality compared to refined grains by increasing potassium and fiber intake (51). Taking into account the nutritional facts of potatoes, however, it must be noted that increasing potassium intake tends to be more clinically relevant than increasing fiber intake. For example, according to the USDA, there are 372 mg of potassium and 1.4 g of total fiber per 100 g of white boiled potato [FDC ID: 1102882 (52)], and optimal intakes of potassium are at ~3000 mg/d (53) and of fiber at ~25-38 g/d (53).

It is noteworthy that the interest in developing pharmacological agents capable of converting meals into low GI meals, i.e., alpha-glucosidase inhibitors (acarbose and voglibose), reinforces the importance of low GI as a means of reducing the risk of T2DM and accompanying diseases by improving glycemic control and post-load insulin levels (54, 55). Regarding the medication use of our population, participants took only oral hypoglycemic agents for diabetes control, and hence those who were on insulin therapy were not included. While our results aim at patients with T2DM on oral hypoglycemic agents, it is imperative to mention that high GI foods can be fundamental to mitigating hypoglycemic episodes in patients with T1DM receiving intensive insulin therapy. Therefore, the use of high GI cannot be considered harmful as a whole; instead, it may be useful in some circumstances.

Notwithstanding the attractive concept of GI, a plethora of dietary strategies (e.g., DASH diet, Mediterranean diet, and intermittent fasting) can assist in metabolic effects regardless of GI (56–60). Albeit a low free sugar diet is an efficient method of reducing hepatic steatosis and fibrosis while improving glycemic indices in patients with NAFLD (61), long-term adherence ought to be considered and thus dietary models with moderate amounts of sugars are apparently more feasible. Interestingly, a recent RCT shows that intermittent fasting can reduce hepatic steatosis alongside fat mass in patients with NAFLD (62), and intermittent fasting has emerged as a flexible dietary model in which moderate amount of sugars are easily considered within a personalized approach. In general, however, energy restriction can be the cornerstone of dietary efficacy irrespective of the pattern (63, 64). Given that the individuals in our study were in the overweight classification, they would appear to have metabolic benefits in case of an energy‐restricted intervention.

Beyond manipulation of carbohydrate intake, different dietary models, and energy restriction, functional foods can be considered in an attempt to control T2DM and NAFLD. For instance, omega-3 fatty acids are essential nutrients that can be part of the treatment of both diseases (65). Furthermore, vinegar, cinnamon, curcumin, garlic, and ginger are some examples of common functional foods that can improve the metabolism of glucose and TG (66–73). The latter items are sources of antioxidants, which may attenuate the formation of advanced glycation end products and therefore provide benefits in metabolic and tissue markers (74, 75). Although promising, there is no consensus regarding the use of natural products as a first-line treatment to cure/alleviate NAFLD, whose physicians should only consider them an alternative therapeutic approach (76). At last, the practice of physical exercise is another non-pharmacological approach that deserves substantial attention because of its undisputable effects on improving glucose update and reducing visceral fat (77).

Our study has limitations that cannot be neglected. First and foremost, we did not have access to the types of hypoglycemic agents and hence we did not control the statistical confounders for medications. Indeed, the control for hypoglycemic agents is crucial, as some agents provide benefits to NAFLD progression, but at different magnitudes (78). Second, the cross-sectional design does not assess causality due to the lack of temporality. Third, the sample size can be questioned when compared with epidemiological studies. Fourth, although we used a validated FFQ, remembering the frequencies of food consumption during the last year is a limitation of FFQs. Lastly, people with T2DM had some dietary restrictions which made ranges between GI tertiles very narrow resulting in difficulty in finding differences. Since the present study was conducted in patients with BMI ≥ 23 kg/m^2^, further studies encompassing patients within the traditional healthy BMI range of 18.5 to 24.9 kg/m^2^ are required.

## 5 Conclusion

A low GI diet can decrease the odds of having NAFLD in patients with T2DM. Further interventional and prospective studies are warranted to confirm these findings and investigate causal links.

## Data availability statement

The raw data supporting the conclusions of this article will be made available by the authors, without undue reservation.

## Ethics statement

The present study was approved by the Ethics Committee of the Shahid Beheshti University of Medical Sciences, Tehran, Iran (NO: IR.SBMU.NNFTRI.REC.1399.061). The patients provided their written informed consent to participate in this study.

## Author contributions

MS: Conceptualization, Formal analysis, Writing—Original Draft. SS and AM: Investigation, Resources. AAK and HS: Writing—Original Draft. ZA and HK-V: Writing—Review & Editing. HP: Data Curation. MK, FA-S, HS, and AH: Writing—Review & Editing. AH: Supervision. All authors have read and approved the final version to be published.
